# Role of the Peroxisome Proliferator-Activated Receptors, Adenosine Monophosphate-Activated Kinase, and Adiponectin in the Ovary

**DOI:** 10.1155/2008/176275

**Published:** 2007-08-24

**Authors:** Joëlle Dupont, Christine Chabrolle, Christelle Ramé, Lucie Tosca, Stéphanie Coyral-Castel

**Affiliations:** Unité de Physiologie de la Reproduction et des Comportements, Institut National de la Recherche Agronomique, 37380 Nouzilly, France

## Abstract

The mechanisms controlling the interaction between energy balance and reproduction are the subject of intensive investigations. The integrated control of these systems is probably a multifaceted phenomenon involving an array of signals governing energy homeostasis, metabolism, and fertility. Two fuel sensors, PPARs, a superfamily of nuclear receptors and the kinase AMPK, integrate energy control and lipid and glucose homeostasis. Adiponectin, one of the adipocyte-derived factors mediate its actions through the AMPK or PPARs pathway. These three molecules are expressed in the ovary, raising questions about the biological actions of fuel sensors in fertility and the use of these molecules to treat fertility problems. This review will highlight the expression and putative role of PPARs, AMPK, and adiponectin in the ovary, particularly during folliculogenesis, steroidogenesis, and oocyte maturation.

## 1. INTRODUCTION

The levels of various molecules, including metabolites (glucose, fatty acids, amino acids) and hormones (adiponectin, insulin, leptin, ghrelin, etc.), are modulated by nutrition and energy supply. Most of these molecules are known to be directly involved, through a fuel sensor, in the regulation of fertility at each level of the hypothalamo-pituitary-gonad axis (for review see [1, 2]). For example, mice lacking insulin-signalling pathway components, such as insulin receptor substrate 2 (IRS-2) or insulin receptor, display female and male infertility [3, 4].

In humans, a close link between energy status and reproductive function has been found in some diseases. Polycystic ovary syndrome (PCOS), which is frequently associated with insulin resistance, affects 5 to 10% of women of reproductive age [5]. Women with PCOS present with ovulation problems, which may be associated with infertility. The treatment of PCOS patients with insulin-sensitising agents of various drug families, such as thiazolidinediones (TZDs) or metformin (a derivative of biguanide), restores the menstrual cycle [6] and increases ovulation (by improving follicular growth), fertilization, and pregnancy rates [7]. TZDs bind to the nuclear peroxisome proliferator-activated receptor gamma (PPARγ) and metformin activates the AMP-activated protein kinase (AMPK) pathway [8, 9]. In women with PCOS, plasma adiponectin is also significantly decreased independently of obesity [10]. Adiponectin plasma levels seem to be related to TZDs or Metformin treatment. Adiponectin is an adipokine known to increase sensitivity to insulin and vasodilatation (for review [11]). Adiponectin could also be involved in the regulations of some reproductive functions [12, 13]. In mammals, and particularly in cattle, dietary fats also influence reproductive function. For example, fatty acid supplementation in the diet increases the total number of follicles and stimulates growth of the preovulatory follicle [14]. In cows, the availability of fatty acid precursors is coupled with an increase in sexual steroid levels and eicosanoid secretion, potentially affecting ovarian and uterine function and embryo implantation [15]. These phenomena may involve several hormones including insulin, IGFs, leptin, adiponectin, and some factors such as PPARs and AMPK. Indeed, these molecules are known to play a role in energy control and lipid metabolism. They may hypothetically play a role as fuel sensors in reproductive compartments, providing the cells with information about energy status. However, how metformin and TZDs influence ovarian function is still under investigation. The functions of PPARs, AMPK, and adiponectin in the ovary also remain unclear. In this review, we will describe the expression and potential implications of these fuel sensors in the ovary.

## 2. PPARs AND AMPK STRUCTURES AND IMPLICATIONS

The PPAR family (α, β/δ and γ) integrates energy control with lipid and glucose metabolism and affects insulin sensitivity [16]. Like PPARs, AMPK plays a key role in regulating lipid and glucose metabolism in response to metabolic stress and energy demand [17]. AMPK acts at various steps and plays a central role in controlling fatty acid, triglyceride, and cholesterol synthesis, and the oxidation of fatty acids, through direct phosphorylation and control over gene transcription [17].

PPARs and AMPK have similar effects and close links have been found between these molecules. Indeed, it is generally assumed that TZDs activate PPARγ and AMPK independently [18–20]. The inhibition of AMPK expression by siRNA abolishes the inhibitory effects of rosiglitazone and 15d-PGJ_2_ (two PPARγ ligands, see below) on iNOS expression and activity [21]. The mitochondria may house a pathway common to PPARγ and AMPK. Indeed, both metformin and TZDs cause a rapid increase in cellular ADP : ATP ratio, probably by inhibiting the respiratory chain, leading to the phosphorylation and activation of AMPK [22]. PPARs and AMPK also participate in the molecular action of adiponectin, an adipocytokine involved in the insulin sensitivity of tissues [7].

### 2.1. Structure and mechanisms of action of PPARs

The PPARs are transcription factors that share a common structure with steroid hormone receptors: the N-terminal A/B domain responsible for ligand-independent transactivation function, the C domain containing the DNA-binding domain, the D domain involved in the receptor dimerization, and the C-terminal E/F domain containing the ligand binding domain (for review [23]). The members of the nuclear PPAR (α, β/δ, and γ) family bind to specific regions of DNA in heterodimers with the retinoid X receptors (RXRs) [24]. These DNA sequences are known as PPREs (peroxisome proliferator response elements). The transcription is activated subsequent to heterodimerisation of PPAR and retinoid receptors (RXR). Furthermore, PPARs are able to indirectly regulate gene expression through transrepression mechanisms by linking some cofactors (reviewed in [23]). In this review, we focus on the PPARα and PPARγ isoforms.

The stimulation of PPARγ by TZDs modifies the transcription and/or the activity of several key regulators of energy homeostasis, including several glucose regulators (glucose transporters, insulin receptor, IRS, etc.), which it stimulates (for review see [25, 26]). PPARs regulate the transcription of a number of target genes involved in ovarian functions such as steroidogenesis, ovulation, oocyte maturation, and maintenance of the corpus luteum (cyclooxygenase-2 (COX-2), nitric oxide synthase (NOS), several proteases, including matrix metalloprotease-9, plasminogen activator, and vascular endothelial growth factor (VEGF), reviewed in [23]). PPARγ activity is governed by binding to small lipophilic ligands, such as polyunsaturated fatty acids and eicosanoids derived from the diet or metabolic pathways (e.g., the prostaglandin D2 metabolite 15-deoxy-12, 14-prostanglandin J2 (PGJ_2_)) [27]. PPARγ is also activated by synthetic compounds called thiazolidinediones (TZDs), a class of insulin-sensitising agents. PPARγ may also be regulated by AMPK. Indeed, AMPK can phosphorylate PPARγ, repressing both the ligand-dependent and ligand-independent transactivating functions of this receptor [28].

PPARα is another isoform of PPAR expressed in the ovary. It regulates genes responsible for the uptake into cells and beta-oxidation of fatty acids [29]. Hypolipidaemic fibrate drugs, phthalate esters (plasticisers, herbicides), and long-chain polyunsaturated fatty acids and their lipooxygenase-derived metabolites (e.g., leukotriene) have been described as agonists of PPARα [30–32]. In vivo, fibrates are currently administrated alone or in combination with statins to patients with increased cardiovascular risk to impede the progression of atherosclerotic lesions. Insulin increases the transcriptional activity of PPARα by activating the MAPK pathway [33]. New therapeutics agents, such as glitazar, may activate both PPARα and PPARγ [34].

### 2.2. Structure and mechanisms of action of AMPK

Unlike PPARs, AMPK is a kinase comprised of three subunits: a catalytic subunit alpha and two regulatory subunits, beta and gamma [35]. The alpha subunit contains the catalytic core and binds, via its C-terminal tail, to the beta subunit, which serves as a docking subunit for the alpha and gamma subunits. AMPK is activated by a change in the AMP : ATP ratio within the cell and therefore acts as an efficient sensor of cellular energy state. This change in AMP : ATP ratio may result from exercise [36], hypoxia [37], hormones [38, 39], or the effects of pharmacological drugs, such as 5-aminoimidazole-4-carboxamide-riboside-5-phosphate (AICAR) [40]. Binding to AMP activates AMPK allosterically and induces phosphorylation of the threonine 172 residue of the α subunit by upstream kinases, including the tumour suppressor LKB1 [41, 42].

AMPK phosphorylates target proteins (including PPARγ) involved in a number of metabolic pathways, including lipid and cholesterol metabolism (adipocytes, liver, and muscle), glucose transport, glycogen, and protein metabolism (see review [35, 41]).

### 2.3. Involvement of PPARs and AMPK in the adiponectin action

**Figure 1:**
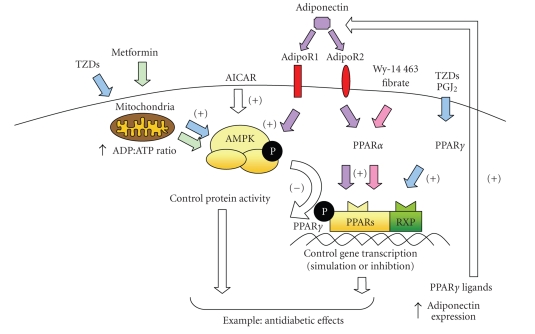
Schema illustrating the putative functional interactions between PPARs, AMPK, and adiponectin. PPARγ is activated by binding with PGJ_2_ or TZDs and PPARα with fibrates or WY 14 463. They control gene transcription, and, in particular, PPARγ ligands increase adiponectin expression [49]. Metformin and TZDs activate AMPK probably via the respiratory chain in mitochondria [22], and AICAR stimulates AMPK. AMPK controls protein activity by phosphorylation (e.g., inhibits PPARγ by phosphorylation [35]). Adiponectin activates AdipoR1 and AdipoR2 receptors which act on metabolism via AMPK (AdipoR1) or PPARα (AdipoR2) [43].

AMPK and PPARα are both activated by adiponectin [11, 43] (Figure 1). Adiponectin (also known as apM1, AdipoQ, Gbp28, and Acrp30) is an adipocyte-derived factor [44, 45]. It is present as a multimer at high concentrations in the circulation (5 to 25 *μ*g/ml in human [46]). In obese and type 2 diabetic humans, plasma adiponectin is strongly reduced suggesting that circulating adiponectin may be related to the development of insulin resistance [11]. Two adiponectin receptors (AdipoR1 and AdipoR2) have been identified in different tissues of various species. They each contain seven transmembrane domains, but are structurally and functionally different from G protein-coupled receptors. Adiponectin plays an important role in insulin sensitisation in mammals (inhibition of gluconeogenesis and stimulation of fatty acid oxidation) by activating AMPK [47] and PPARα proteins in skeletal muscle, liver, and adipocytes [43]. Thus, both TZDs and adiponectin have been shown to activate AMPK. Moreover, the promoter of the adiponectin gene contains a PPRE [48] and TZDs increase the production and plasma concentration of adiponectin [49]. TZDs have weaker antidiabetic effects in ob/ob mice lacking adiponectin gene than in ob/ob mice with adiponectin, and the activation of AMPK by TZDs is also attenuated in these mice, suggesting that adiponectin is required for the activation of AMPK by TZDs [50].

In porcine granulosa cells, adiponectin treatment induces the expression of genes associated with periovulatory remodeling of the ovarian follicle (cyclooxygenase-2, prostaglandin E synthase, and vascular endothelial growth factor [51]). Some of these genes are also activated by PPARγ. Furthermore, adiponectin receptors, PPARs, and AMPK are expressed in reproductive tissues, including the ovary.

## 3. EXPRESSION OF PPARs AND AMPK IN THE OVARY

### 3.1. Expression of PPARs in the ovary

All the PPAR isoforms are expressed in the ovary. The PPARα and PPARβ/δ isoforms are expressed primarily in the theca and stroma tissues [52], reviewed by [23], (see Table 1). The deletion of PPARα has no apparent effect on the fertility of mice, whereas PPARβ/δ-null mice present placental malformations leading to embryo death during early pregnancy [53–55]. PPARγ is expressed strongly in granulosa cells, and less strongly in the theca cells and corpus luteum, in the ovaries of rodents and ruminants (see Table 1) [52, 56, 57]. PPARγ is detected early in folliculogenesis (at the primary/secondary follicle stage) [58], and its expression increases until the large follicle stage and then decreases after the LH surge [58]. In mice, the absence of PPARγ in the ovaries results in lower levels of fertility [59]. No effect on folliculogenesis or ovulation rate has been observed, but fewer embryos implant, probably due to lower levels of progesterone production by the corpus luteum [59].

### 3.2. Expression of AMPK and adiponectin in the ovary

AMPK expression has been studied in the ovaries of various species, including rat [60, 65], mouse [61], cow [62], pig [63], and chicken [64]. RT-PCR has shown the mRNAs of all the AMPK subunits to be present in granulosa cells, the corpus luteum, oocyte, and cumulus-oocyte-complexes in rodent and bovine ovaries (Table 1) [60, 62]. We have shown, by immunohistochemical analyses, that the AMPK α-subunit, like PPARγ, is more strongly expressed in granulosa cells than in theca cells in rats and cows [60, 62]. In cows, levels of AMPKα- and β-subunits were similar in small and large follicles. In hens, the activation of AMPK by its phosphorylation on the Thr172 residue increased during follicle development [64]. In mice, the absence of the catalytic AMPK alpha 2 subunit does not affect female fertility [66]. Until now, no data are available on the reproductive functions of the transgenic or knockout mice for the other subunits of AMPK.

In chicken ovary, adiponectin mRNA is more abundant in theca cells than in granulosa cells (Table 1) [13]. In porcine ovary, adiponectin is detected at similar concentrations in the follicular fluid and serum [51]. Both receptors are expressed in ovarian follicles. In chicken, the adiponectin type I receptor (AdipoRI) is twice as abundant in granulosa cells as in theca cells, and the type II receptor (AdipoR2) is expressed equally strongly in granulosa and thecal cells (Table 1) [13]. Studies in mice have shown that AdipoR1 may be more tightly linked to AMPK pathway activation, whereas AdipoR2 seems to be associated with PPARα activation [43]. However, mice lacking adiponectin [67], AdipoR1, AdipoR2, or both receptors [43] are fertile, which suggests that this signalling is not absolutely essential for ovarian function. However, it may be required for ovulation in other species or may simply be an additional component for fine-tuning ovarian function.

## 4. FUNCTION OF PPARs, AMPK, AND ADIPONECTIN IN THE OVARY

### 4.1. Regulation of steroidogenesis by PPARγ, PPARα, AMPK, and adiponectin

TZDs modulate cell proliferation and steroidogenesis in granulosa cells in vitro (reviewed by [23]). Sex steroid secretion (progesterone, oestradiol) may be inhibited by TZDs in sows and in women undergoing in vitro fertilization [56, 68] or stimulated (progesterone and oestradiol), as in rats and ewes [52, 57]). The effects of TZDs depend on the species and the status of granulosa cell differentiation (follicular phase, before or after the gonadotropin surge in human granulosa cells). TZDs could regulate their target genes at the transcriptional level (reviewed by [23, 68]). However, several studies have suggested that TZDs could also exert their effects by modifying the activity of steroidogenic enzymes (3-beta-hydroxysteroid-dehydrogenase (3-βHSD) and aromatase) [56, 69]. Indeed, the concentrations of Cyp11a1 and 3-βHSD mRNA in porcine granulosa cells and the levels of the corresponding proteins in ovine granulosa cells are not affected by TZD treatment [56, 57]. Moreover, TZDs increase the release of pregnenolone, a substrate of 3β-HSD, from porcine granulosa cells into the medium, whereas progesterone production decreases [56]. Ligands for PPARα are also known to alter ovarian steroidogenesis. For example, in vivo. fenofibrate, through PPARα-dependent mechanism, inhibits aromatase cytochrome P450 expression and activity in the ovary of mouse [70]. Another PPARα synthetic ligand, Wy-14 463, suppresses also aromatase transcript levels and oestradiol production in cultured rat granulosa cells [71]. 

AMPK, like PPARγ and PPARα, may influence ovarian function by modifying the synthesis of progesterone and oestradiol. Studies based on AICAR and the adenovirus-mediated expression of dominant negative AMPK have demonstrated that AMPK reduces progesterone production, but not oestradiol production, in rat granulosa cells [60]. This decrease is associated with a decrease in 3β-HSD mRNA and protein levels and a decrease in MAPK ERK1/2 phosphorylation [60]. Furthermore, the activation of AMPK by metformin decreases basal and FSH-induced progesterone secretion by decreasing the levels of proteins involved in steroidogenesis: (3βHSD, CYP11a1, STAR) [65]. In granulosa cells from humans and cows, metformin strongly decreases the secretion of progesterone and oestradiol [62, 72]. In bovine granulosa cells, this effect is mediated by AMPK activation, and leads to a decrease in MAPK activation. In human granulosa cells, metformin also decreases androgen synthesis, by directly inhibiting Cyp17 activity [73]. Thus, AMPK activation decreases steroidogenesis in the granulosa cells of various species. The effects of AMPK on steroid secretion, like those of PPARγ, depend on the species and the stimulator of AMPK (AICAR versus metformin). Several results suggest that metformin-induced AMPK activation could act through transcriptional mechanism. Further investigations are needed to determine the molecular mechanism of metformin.

**Figure 2:**
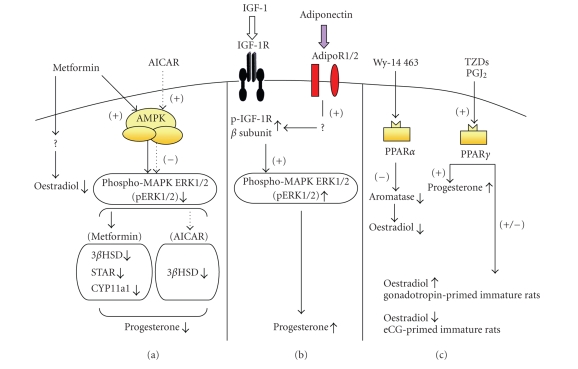
Schema illustrating the effects of (a) metformin- or AICAR-induced AMPK activation, (b)adiponectin, and (c) TZDs or PPAR alpha ligands on the rat granulosa cell steroidogenesis. (a) Metformin or AICAR treatment decreases MAPK ERK1/2 phosphorylation and progesterone secretion through AMPK activation [60, 65]. Metformin decreases also oestradiol secretion through an independent AMPK pathway [60]. (b) Adiponectin treatment increases IGF-1-induced IGF-1R β-subunit tyrosine phosphorylation and MAPK ERK1/2 phosphorylation and progesterone secretion [12]. (c) The PPARα ligand, Wy-14 463, inhibits oestradiol secretion whereas TZDs or PGJ2 increases progesterone secretion and inhibits estradiol secretion in eCG-primed immature rats or increases estradiol secretion in gonadotropin-primed immature rat [23, 52]. 3βHSD: 3β-hydroxysteroiddehydrogenase, STAR: Steroidogenic acute regulatory protein, CYP11a1: P450 sidechain cleavage, Adipo R1/2: Adiponectin receptor type I and II, MAPK ERK1/2: Mitogen Activated protein kinase Extracellular Regulated kinase, 1/2, PGJ2: prostaglandine J2.

Women treated for in vitro fertilization (IVF) present an increase in serum adiponectin concentration after the administration of human chorionic gonadotropin, this increase being correlated with progesterone levels [74]. In cultured porcine granulosa cells, adiponectin modulates the expression of genes encoding proteins involved in steroid production, increasing the abundance of transcripts for the steroidogenic acute regulatory protein, and decreasing the abundance of cytochrome P450 aromatase transcripts [51]. The MAPK pathway, rather than protein kinase A or AMPK, mediates the adiponectin signal in ovarian granulosa cells, by ERK1/2 phosphorylation [51]. Surprisingly, adiponectin alone does not affect steroid production in rat granulosa cells [12]. However, it approximately doubled the IGF-1-induced secretion of progesterone. These effects may be due to an increase in IGF-1 receptor beta subunit tyrosine phosphorylation and ERK1/2 phosphorylation [12]. A schema illustrating the effects of PPARα and γ, AMPK and adiponectin activation on the steroidogenesis of rat granulosa cells is shown in Figure 2.

### 4.2. Regulation of granulosa cell proliferation

In addition to their effects on steroidogenesis, TZDs decrease the proliferation of granulosa cells in sheep (PPARγ, [57]). These data are in good agreement with those obtained in bovine lutein cells since an aurintricarboxylic acid-mediated decrease of PPARγ is accompanied by a progression of the cell cycle [75]. In our knowledge, there are no data on the effects of PPARα ligands on granulosa cell proliferation. In contrast, AMPK and adiponectin are not essential for granulosa cell proliferation in rat [12, 60].

### 4.3. Regulation of oocyte maturation

PPARγ, AMPK, and adiponectin are all expressed in mammalian oocytes [12, 23, 60, 76]. However, AMPK has been studied in more detail than PPARγ, PPARα, and adiponectin. PPARγ may regulate the expression of genes involved in the meiotic maturation of oocytes (e.g., nitric oxide synthase (NOS)) [23]. Wood et al. recently identified putative binding sites for PPARγ/RXR in the proximal promoters of several genes differentially expressed in oocytes from women with PCOS and known to play a role in the meiotic cell cycle [77]. All these results suggest that PPARγ/RXR may be active in the oocyte. The two adiponectin receptors, AdipoR1 and AdipoR2, are also expressed in rat oocytes, and AMPK activity has also been detected in oocytes of several species (see above), suggesting that adiponectin may play a role through AMPK in determining oocyte quality (cited by [78]). In addition, women with PCOS showing impairment in the final maturation of oocytes and in ovulation, present lower circulating concentrations of adiponectin [10, 79].

In vivo, the oocyte remains at the immature stage or germinal vesicle stage (GV, i.e., prophase of meiosis I) until the preovulatory LH surge [79]. However, if cumulus-oocyte complexes (COCs) are removed from the follicles and cultured in vitro, oocytes may spontaneously resume meiosis [80, 81]. During nuclear maturation, immature oocytes undergo germinal vesicle breakdown (GVBD) and proceed through metaphase II of meiosis. The pharmacological activation of AMPK, by AICAR injection, in mouse oocytes leads to the induction of oocyte maturation in arrested cumulus-enclosed oocytes [82]. Metabolic stresses (oxidative or osmotic) known to activate AMPK accelerate meiosis in oocytes in which meiosis was previously arrested by cAMP analogues [83]. However, the data for mice conflict with those obtained with porcine and bovine oocytes [84, 85]. Indeed, in these two latter species, AICAR and metformin significantly increase phosphorylation/activation of AMPK and the percentage of COCs arrested at the GV stage. Thus, AMPK activation has opposite effects in the control of oocyte maturation in cows, sows and mice. This could be explained by the important differences that exist in the regulation of oocyte meiotic resumption between rodent and nonrodent animals such as for example the time taken for oocytes to undergo meiotic resumption (2 to 3 hours of in vitro maturation in rodent, 20 hours in pig, and 22 hours in bovine species). Interestingly, in women with PCOS treated with metformin, the number of mature oocytes retrieved and oocytes fertilized has been shown to increase after gonadotropin stimulation for IVF [86]. However, recent data indicate that clomiphene is superior to metformin in achieving live birth in infertile women with PCOS [87].

## 5. CONCLUSION

The nuclear PPARs and the fuel sensor AMPK are expressed in the ovary of various species. Several studies have shown that they modulate ovarian cell proliferation and steroidogenesis and could be involved in oocyte maturation. Both PPARα and AMPK mediate the effects of hormones involved in lipid and glucose metabolism, including adiponectin. Thus, PPARs, AMPK, and adiponectin may be key signals regulating the amount of energy required for the growth of follicles, oocytes, and embryos. Further investigations are necessary to assess the exact importance and mechanisms of action of these molecules in some ovarian dysfunctions including for example PCOS syndrome.

## Figures and Tables

**Table 1:** Location of PPARs, AMPK, and adiponectin in ovary.

	Species	Location	mRNA or Protein	References
PPARα	Rat	Theca and stroma		[52]

PPARβ/δ	Rat	Throughout the ovary		[52]

PPARγ	Mouse, rat, pig, sheep, cow, and human	Granulosa, corpus, luteum, porcine theca and granulosa cells oocytes		Reviewed by [23]

AMPK	Rat, cow, chicken, pig, mouse	Granulosa cells, oocyte, corpus luteum (weaker in rat theca cells for AMPK α1)	mRNA and protein	[60–64]

Adiponectin	Rat, chicken, pig	Theca cells, oocyte, and corpus luteum, Follicular liquid	mRNA (chicken) mRNA and protein (rat)	[12, 13, 51]

Adiponectin receptor I	Rat, chicken, pig	Granulosa and theca cells, oocyte and corpus luteum (rat)	mRNA (chicken) mRNA and protein (rat)	[12, 13, 51]

Adiponectin receptor II	Rat, chicken, pig	Granulosa cells, oocyte and corpus luteum (rat)	mRNA (chicken) mRNA and protein (rat)	[12, 13, 51]

## References

[1] Butler WR (2000). Nutritional interactions with reproductive performance in dairy cattle. *Animal Reproduction Science*.

[2] Fernandez-Fernandez R, Martini AC, Navarro VM (2006). Novel signals for the integration of energy balance and reproduction. *Molecular and Cellular Endocrinology*.

[3] Burks DJ, de Mora JF, Schubert M (2000). IRS-2 pathways integrate female reproduction and energy homeostasis. *Nature*.

[4] Bruning JC, Gautam D, Burks DJ (2000). Role of brain insulin receptor in control of body weight and reproduction. *Science*.

[5] Dunaif A (1997). Insulin resistance and the polycystic ovary syndrome: mechanism and implications for pathogenesis. *Endocrine Reviews*.

[6] Iuorno MJ, Nestler JE (2001). Insulin-lowering drugs in polycystic ovary syndrome. *Obstetrics and Gynecology Clinics of North America*.

[7] Seli E, Duleba AJ (2004). Treatment of PCOS with metformin and other insulin-sensitizing agents. *Current Diabetes Reports*.

[8] Lehmann JM, Moore LB, Smith-Oliver TA, Wilkison WO, Willson TM, Kliewer SA (1995). An antidiabetic thiazolidinedione is a high affinity ligand for peroxisome proliferator-activated receptor γ (PPARγ). *Journal of Biological Chemistry*.

[9] Musi N, Hirshman MF, Nygren J (2002). Metformin increases AMP-activated protein-kinase activity in skeletal muscle of subjects with type 2 diabetes. *Diabetes*.

[10] Ardawi MS, Rouzi AA (2005). Plasma adiponectin and insulin resistance in women with polycystic ovary syndrome. *Fertility and Sterility*.

[11] Kadowaki T, Yamauchi T (2005). Adiponectin and adiponectin receptors. *Endocrine Reviews*.

[12] Chabrolle C, Tosca L, Dupont J (2007). Regulation of adiponectin and its receptors in rat ovary by human chorionic gonadotrophin treatment and potential involvement of adiponectin in granulosa cell steroidogenesis. *Reproduction*.

[13] Chabrolle C, Tosca L, Crochet S, Tesseraud S, Dupont J Expression of adiponectin and its receptors (AdipoR1 and AdipoR2) in chicken ovary: potential role in ovarian steroidogenesis.

[14] Mattos R, Staples CR, Thatcher WW (2000). Effects of dietary fatty acids on reproduction in ruminants. *Reviews of Reproduction*.

[15] Garcia-Bojalil CM, Staples CR, Risco CA, Savio JD, Thatcher WW (1998). Protein degradability and calcium salts of long-chain fatty acids in the diets of lactating dairy cows: reproductive responses. *Journal of Dairy Science*.

[16] Kota BP, Huang TH-W, Roufogalis BD (2005). An overview on biological mechanisms of PPARs. *Pharmacological Research*.

[17] Hardie DG, Carling D (1997). The AMP-activated protein kinase—fuel gauge of the mammalian cell?. *European Journal of Biochemistry*.

[18] Han S, Roman J (2006). Rosiglitazone suppresses human lung carcinoma cell growth through PPARγ-dependent and PPARγ-independent signal pathways. *Molecular Cancer Therapeutics*.

[19] LeBrasseur NK, Kelly M, Tsao T-S (2006). Thiazolidinediones can rapidly activate AMP-activated protein kinase in mammalian tissues. *American Journal of Physiology*.

[20] Fryer LG, Parbu-Patel A, Carling D (2002). The anti-diabetic drugs rosiglitazone and metformin stimulate AMP-activated protein kinase through distinct signaling pathways. *Journal of Biological Chemistry*.

[21] Pilon G, Dallaire P, Marette A (2004). Inhibition of inducible nitric-oxide synthase by activators of AMP-activated protein kinase: a new mechanism of action of insulin-sensitizing drugs. *Journal of Biological Chemistry*.

[22] Owen MR, Doran E, Halestrap AP (2000). Evidence that metformin exerts its anti-diabetic effects through inhibition of complex 1 of the mitochondrial respiratory chain. *Biochemical Journal*.

[23] Komar CM (2005). Peroxisome proliferator-activated receptors (PPARs) and ovarian function—implications for regulating steroidogenesis, differentiation, and tissue remodeling. *Reproductive Biology and Endocrinology*.

[24] Miyata KS, McCaw SE, Marcus SL, Rachubinski RA, Capone JP (1994). The peroxisome proliferator-activated receptor interacts with the retinoid X receptor in vivo. *Gene*.

[25] Desvergne B, Wahli W (1999). Peroxisome proliferator-activated receptors: nuclear control of metabolism. *Endocrine Reviews*.

[26] Picard F, Auwerx J (2002). PPARγ and glucose homeostasis. *Annual Review of Nutrition*.

[27] Kobayashi Y, Ueki S, Mahemuti G (2005). Physiological levels of 15-deoxy-$\Delta^{12,14}$-prostaglandin ${\text{J}}_{2}$ prime eotaxin-induced chemotaxis on human eosinophils through peroxisome proliferator-activated receptor-γ ligation. *Journal of Immunology*.

[28] Leff T (2003). AMP-activated protein kinase regulates gene expression by direct phosphorylation of nuclear proteins. *Biochemical Society Transactions*.

[29] Braissant O, Foufelle F, Scotto C, Dauça M, Wahli W (1996). Differential expression of peroxisome proliferator-activated receptors (PPARs): tissue distribution of PPAR-α, -β, and -γ in the adult rat. *Endocrinology*.

[30] Krey G, Braissant O, L'Horset F (1997). Fatty acids, eicosanoids, and hypolipidemic agents identified as ligands of peroxisome proliferator-activated receptors by coactivator-dependent receptor ligand assay. *Molecular Endocrinology*.

[31] Schoonjans K, Staels B, Auwerx J (1996). Role of the peroxisome proliferator-activated receptor (PPAR) in mediating the effects of fibrates and fatty acids on gene expression. *Journal of Lipid Research*.

[32] Zhou Y-C, Waxman DJ (1998). Activation of peroxisome proliferator-activated receptors by chlorinated hydrocarbons and endogenous steroids. *Environmental Health Perspectives*.

[33] Shalev A, Siegrist-Kaiser CA, Yen PM (1996). The peroxisome proliferator-activated receptor α is a phosphoprotein: regulation by insulin. *Endocrinology*.

[34] Fiévet C, Fruchart J-C, Staels B (2006). PPARα and PPARγ dual agonists for the treatment of type 2 diabetes and the metabolic syndrome. *Current Opinion in Pharmacology*.

[35] Hardie DG (2004). The AMP-activated protein kinase pathway—new players upstream and downstream. *Journal of Cell Science*.

[36] Hayashi T, Hirshman MF, Kurth EJ, Winder WW, Goodyear LJ (1998). Evidence for 5' AMP-activated protein kinase mediation of the effect of muscle contraction on glucose transport. *Diabetes*.

[37] Mu J, Brozinick JT, Valladares O, Bucan M, Birnbaum MJ (2001). A role for AMP-activated protein kinase in contraction- and hypoxia-regulated glucose transport in skeletal muscle. *Molecular Cell*.

[38] Yamauchi T, Kamon J, Minokoshi Y (2002). Adiponectin stimulates glucose utilization and fatty-acid oxidation by activating AMP-activated protein kinase. *Nature Medicine*.

[39] Minokoshi Y, Kim Y-B, Peroni OD (2002). Leptin stimulates fatty-acid oxidation by activating AMP-activated protein kinase. *Nature*.

[40] Corton JM, Gillespie JG, Hawley SA, Hardie DG (1995). 5-aminoimidazole-4-carboxamide ribonucleoside—a specific method for activating AMP-activated protein kinase in intact cells?. *European Journal of Biochemistry*.

[41] Kahn BB, Alquier T, Carling D, Hardie DG (2005). AMP-activated protein kinase: ancient energy gauge provides clues to modern understanding of metabolism. *Cell Metabolism*.

[42] Hardie DG (2005). New roles for the LKB1→AMPK pathway. *Current Opinion in Cell Biology*.

[43] Yamauchi T, Nio Y, Maki T (2007). Targeted disruption of AdipoR1 and AdipoR2 causes abrogation of adiponectin binding and metabolic actions. *Nature Medicine*.

[44] Scherer PE, Williams S, Fogliano M, Baldini G, Lodish HF (1995). A novel serum protein similar to C1q, produced exclusively in adipocytes. *Journal of Biological Chemistry*.

[45] Maeda K, Okubo K, Shimomura I, Funahashi T, Matsuzawa Y, Matsubara K (1996). cDNA cloning and expression of a novel adipose specific collagen-like factor, apM1. *Biochemical and Biophysical Research Communications*.

[46] Pajvani UB, Scherer PE (2003). Adiponectin: systemic contributor to insulin sensitivity. *Current Diabetes Reports*.

[47] Yamauchi T, Kamon J, Minokoshi Y (2002). Adiponectin stimulates glucose utilization and fatty-acid oxidation by activating AMP-activated protein kinase. *Nature Medicine*.

[48] Iwaki M, Matsuda M, Maeda N (2003). Induction of adiponectin, a fat-derived antidiabetic and antiatherogenic factor, by nuclear receptors. *Diabetes*.

[49] Maeda N, Takahashi M, Funahashi T (2001). PPARγ
ligands increase expression and plasma concentrations of adiponectin, an adipose-derived protein. *Diabetes*.

[50] Nawrocki AR, Rajala MW, Tomas E (2006). Mice lacking adiponectin show decreased hepatic insulin sensitivity and reduced responsiveness to peroxisome proliferator-activated receptor γ
agonists. *Journal of Biological Chemistry*.

[51] Ledoux S, Campos DB, Lopes FL, Dobias-Goff M, Palin M-F, Murphy BD (2006). Adiponectin induces periovulatory changes in ovarian follicular cells. *Endocrinology*.

[52] Komar CM, Braissant O, Wahli W, Curry TE (2001). Expression and localization of PPARs in the rat ovary during follicular development and the periovulatory period. *Endocrinology*.

[53] Lee SS, Pineau T, Drago J (1995). Targeted disruption of the α
isoform of the peroxisome proliferator-activated receptor gene in mice results in abolishment of the pleiotropic effects of peroxisome proliferators. *Molecular and Cellular Biology*.

[54] Barak Y, Liao D, He W (2002). Effects of peroxisome proliferator-activated receptor δ
on placentation, adiposity, and colorectal cancer. *Proceedings of the National Academy of Sciences of the United States of America*.

[55] Peters JM, Lee SS, Li W (2000). Growths, adipose, brain, and skin alterations resulting from targeted disruption of the mouse peroxisome proliferator-activated receptor β(δ). *Molecular and Cellular Biology*.

[56] Gasic S, Bodenburg Y, Nagamani M, Green A, Urban RJ (1998). Troglitazone inhibits progesterone production in porcine granulosa cells. *Endocrinology*.

[57] Froment P, Fabre S, Dupont J (2003). Expression and functional role of peroxisome proliferator-activated receptor-γ in ovarian folliculogenesis in the sheep. *Biology of Reproduction*.

[58] Komar C Initiation of peroxysome proliferator-activated receptor gamma (PPARg) expression in the neonatal rat ovary.

[59] Cui Y, Miyoshi K, Claudio E (2002). Loss of the peroxisome proliferation-activated receptor gamma (PPARγ) does not affect mammary development and propensity for tumor formation but leads to reduced fertility. *Journal of Biological Chemistry*.

[60] Tosca L, Froment P, Solnais P, Ferré P, Foufelle F, Dupont J (2005). Adenosine $5^{\prime }$-monophosphate-activated protein kinase regulates progesterone secretion in rat granulosa cells. *Endocrinology*.

[61] Downs SM, Hudson ER, Hardie DG (2002). A potential role for AMP-activated protein kinase in meiotic induction in mouse oocytes. *Developmental Biology*.

[62] Tosca L, Chabrolle C, Uzbekova S, Dupont J (2007). Effects of metformin on bovine granulosa cells steroidogenesis: possible involvement of adenosine $5^{\prime }$monophosphate-activated protein kinase (AMPK). *Biology of Reproduction*.

[63] Mayes MA, Laforest MF, Guillemette C, Gilchrist RB, Richard FJ (2007). Adenosine $5^{\prime }$-monophosphate kinase-activated protein kinase (PRKA) activators delay meiotic resumption in porcine oocytes. *Biology of Reproduction*.

[64] Tosca L, Crochet S, Ferré P, Foufelle F, Tesseraud S, Dupont J (2006). AMP-activated protein kinase activation modulates progesterone secretion in granulosa cells from hen preovulatory follicles. *Journal of Endocrinology*.

[65] Tosca L, Solnais P, Ferré P, Foufelle F, Dupont J (2006). Metformin-induced stimulation of adenosine $5^{\prime }$ progesterone secretion in rat granulosa cells. *Biology of Reproduction*.

[66] Viollet B, Andreelli F, Jørgensen SB (2003). The AMP-activated protein kinase α2 catalytic subunit controls whole-body insulin sensitivity. *Journal of Clinical Investigation*.

[67] Ma K, Cabrero A, Saha PK (2002). Increased β-oxidation but no insulin resistance or glucose intolerance in mice lacking adiponectin. *Journal of Biological Chemistry*.

[68] Froment P, Gizard F, Defever D, Staels B, Dupont J, Monget P (2006). Peroxisome proliferator-activated receptors in reproductive tissues: from gametogenesis to parturition. *Journal of Endocrinology*.

[69] Mu Y-M, Yanase T, Nishi Y (2000). Insulin sensitizer, troglitazone, directly inhibits aromatase activity in human ovarian granulosa cells. *Biochemical and Biophysical Research Communications*.

[70] Toda K, Okada T, Miyaura C, Saibara T (2003). Fenofibrate, a ligand for PPARα, inhibits aromatase cytochrome P450 expression in the ovary of mouse. *Journal of Lipid Research*.

[71] Lovekamp TN, Davis BJ (2001). Mono-(2-ethylhexyl) phthalate suppresses aromatase transcript levels and estradiol production in cultured rat granulosa cells. *Toxicology and Applied Pharmacology*.

[72] Mansfield R, Galea R, Brincat M, Hole D, Mason H (2003). Metformin has direct effects on human ovarian steroidogenesis. *Fertility and Sterility*.

[73] La Marca A, Egbe TO, Morgante G, Paglia T, Ciani A, De Leo V (2000). Metformin treatment reduces ovarian cytochrome P-450c1α7 response to human chorionic gonadotrophin in women with insulin resistance-related polycystic ovary syndrome. *Human Reproduction*.

[74] Liu Y-H, Tsai E-M, Chen Y-L (2006). Serum adiponectin levels increase after human chorionic gonadotropin treatment during in vitro fertilization. *Gynecologic and Obstetric Investigation*.

[75] Löhrke B, Viergutz T, Shahi SK (1998). Detection and functional characterisation of the transcription factor peroxisome proliferator-activated receptor γ
in lutein cells. *Journal of Endocrinology*.

[76] Mohan M, Ryder S, Claypool PL, Geisert RD, Malayer JR (2002). Analysis of gene expression in the bovine blastocyst produced in vitro using suppression-subtractive hybridization. *Biology of Reproduction*.

[77] Wood JR, Dumesic DA, Abbott DH, Strauss JF (2007). Molecular abnormalities in oocytes from women with polycystic ovary syndrome revealed by microarray analysis. *Journal of Clinical Endocrinology and Metabolism*.

[78] Mitchell M, Armstrong DT, Robker RL, Norman RJ (2005). Adipokines: implications for female fertility and obesity. *Reproduction*.

[79] Sir-Petermann T, Echiburú B, Maliqueo MM (2007). Serum adiponectin and lipid concentrations in pregnant women with polycystic ovary syndrome. *Human Reproduction*.

[80] Pincus G, Enzmann EV (1935). The comparative behavior of mammalian eggs in vivo and in vitro. *The Journal of Experimental Medicine*.

[81] Edwards RG (1965). Maturation in vitro of mouse, sheep, cow, pig, rhesus monkey and human ovarian oocytes. *Nature*.

[82] Chen J, Hudson E, Chi MM (2006). AMPK regulation of mouse oocyte meiotic resumption in vitro. *Developmental Biology*.

[83] LaRosa C, Downs SM (2006). Stress stimulates AMP-activated protein kinase and meiotic resumption in mouse oocytes. *Biology of Reproduction*.

[84] Bilodeau-Goeseels S, Sasseville M, Guillemette C, Richard FJ (2007). Effects of adenosine monophosphate-activated kinase activators on bovine oocyte nuclear maturation in vitro. *Molecular Reproduction and Development*.

[85] Tosca L, Uzbekova S, Chabrolle C, Dupont J Possible role of AMPK in the metformin-mediated arrest of bovine oocytes at the GV stage during in vitro maturation.

[86] Stadtmauer LA, Toma SK, Riehl RM, Talbert LM (2002). Impact of metformin therapy on ovarian stimulation and outcome in ‘coasted’ patients with polycystic ovary syndrome undergoing in-vitro fertilization. *Reproductive Biomedicine Online*.

[87] Legro RS, Barnhart HX, Schlaff WD (2007). Clomiphene, metformin, or both for infertility in the polycystic ovary syndrome. *New England Journal of Medicine*.

